# Taking a Detour: Affective Stimuli Facilitate Ultimately (Not Immediately) Compatible Approach–Avoidance Tendencies

**DOI:** 10.3389/fpsyg.2018.00488

**Published:** 2018-04-06

**Authors:** Regina Reichardt

**Affiliations:** Department of Psychology, University of Regensburg, Regensburg, Germany

**Keywords:** approach, avoidance, automatic, ultimate, distal, immediate, affect

## Abstract

Abundant evidence suggests that affective stimuli facilitate responses that lead to a compatible change in distance between the affective stimulus and the self (positive → approach, negative → avoidance). A special situation arises, when a barrier blocks the direct way toward or away from an affective stimulus. Recent evidence suggests that in such cases affective stimuli facilitate responses that ultimately lead to a compatible change in distance, even when this requires an initial step in the opposite and thus incompatible direction. The present study investigated whether this is the case even when relatively complex processing is required to recognize the presence of a barrier and, thus, the need for a detour. Employing a stimulus-response-compatibility task, we asked participants to move a manikin along the pathways of a maze toward or away from a positive or negative stimulus. The direct way was possible on half of the trials and blocked by a barrier on the other half of the trials. In the latter case, the manikin had to first be moved in the direction opposite to the position ultimately intended. We manipulated between participants the type of barrier and, thus, the complexity of cognitive processing required to recognize the need for a detour. In the simple condition, a black bar was presented as a barrier on the way. In the complex condition, a blue or yellow bar was presented, and the color indicated whether the bar constituted a barrier (locked gate) or not (open gate). Replicating and extending previous findings, the present study shows that affective stimuli facilitate ultimately (not immediately) compatible approach–avoidance responses, even when relatively complex processing is required to recognize the need for a detour.

## Introduction

Regulating behavior toward positive and away from negative stimuli is a vital function of living organisms. To fulfill this regulatory need, evaluation of stimulus valence automatically results in the activation of approach and avoidance tendencies (for reviews and meta-analyses, see Phaf et al., 2014; Kozlik et al., 2015; Laham et al., 2015).

To study the automatic impact of affective stimuli on approach–avoidance tendencies, researchers use stimulus-response-compatibility paradigms. In such tasks, participants respond as fast as possible with simple approach and avoidance movements to affective stimuli. For instance, in the widely used manikin task a stick figure (manikin) is presented on a computer screen above or below an affective stimulus shown in the center (De Houwer et al., 2001). Participants move the manikin toward or away from the affective stimulus by pressing the up or down arrow key. Thereby, the compatibility between stimulus valence and response direction is varied. On compatible trials, participants are required to move the manikin toward positive and away from negative stimuli. On incompatible trials, they move the manikin toward negative and away from positive stimuli. Typically, participants are faster to approach positive and avoid negative stimuli than vice versa. According to the rationale underlying this measure, stimulus valence automatically triggers a response tendency (positive → approach, negative → avoidance) that either facilitates (on compatible trials) or interferes (on incompatible trials) with quick responding according to the instructions.

It is important to note that automaticity is not an all-or-none property of cognitive processes. Theoretical accounts distinguish several independent features of automatic processes: fast, efficient, unintentional/goal-independent, and/or unconscious (Bargh, 1992; Moors and De Houwer, 2006). Results from stimulus-response-compatibility paradigms indicate that the impact of stimulus valence on approach–avoidance tendencies is independent of the goal to let stimulus valence influence the responses. That is, because participants are given the goal to respond quickly and accurately independently of the compatibility between stimulus valence and response direction. To test other features of automaticity, researchers have to apply additional manipulations (e.g., adding a response time window, adding cognitive load, or presenting stimuli subliminally). The most widely studied feature of automaticity is independence of evaluation goals. By asking participants to respond to a valence-irrelevant stimulus feature, researchers tested whether approach–avoidance effects depend on the goal to evaluate stimulus valence, however, with mixed results. Even recent meta-analyses come to different conclusions. Whereas Phaf et al. (2014) did not find reliable effects in the absence of evaluation goals, Laham et al. (2015) and Beatty et al. (2016) report significant effects in the absence of evaluation goals, albeit the effects tend to be smaller than when evaluation goals were present (for a detailed discussion, see Reichardt, 2018).

The influence of stimulus valence on approach–avoidance tendencies has been studied with a huge variety of different operationalizations of approach–avoidance behavior. Using a lever, participants were faster to pull positive stimuli toward them and push negative stimuli away from them than vice versa (e.g., Solarz, 1960; Chen and Bargh, 1999). Moreover, affective stimuli facilitated joystick movements that led to a compatible change in the imagined or actual distance between the stimuli and the actual or a virtual self, regardless of whether these movements involved pulling or pushing the joystick (e.g., Markman and Brendl, 2005; Rinck and Becker, 2007; Bamford and Ward, 2008; Seibt et al., 2008). Finally, affective stimuli were shown to facilitate compatible whole-body movements such as taking a step toward or away from the stimuli (Stins et al., 2011).

### Immediate vs. Ultimate Distance Change

Approach and avoidance in real life often require a more complex sequence of behavior than just walking straight toward or away from objects. Sometimes, the direct way is blocked and individuals need to take a detour. For instance, if you look out the window and see a friend approaching your house, you first need to withdraw from the window (and thus from your friend) and go to the door to open it and let your friend in. This detour requires an initial movement in the opposite and thus incompatible direction, i.e., ultimate approach requires initial avoidance. Conversely, there may be situations in which ultimate avoidance may require immediate approach. For instance, if you are afraid of spiders and you want to leave a room because a spider is sitting on the door frame, you need to first approach the spider to ultimately move away from it.

Which behavioral tendencies are automatically activated in such situations, immediate, or ultimate approach–avoidance tendencies? Although there has been extensive research on the automatic activation of approach–avoidance tendencies (Phaf et al., 2014; Laham et al., 2015), this questions has been addressed only in a single publication so far (Krieglmeyer et al., 2011). The authors conducted two studies using two different adapted versions of the manikin task, which allowed them to manipulate whether the direct way was possible or whether a detour was necessary. Overall, the results from both studies suggest that affective stimuli facilitate behavior that ultimately leads to a compatible distance change, even when this requires initially moving in the opposite and thus incompatible direction.

Furthermore, research on oral approach–avoidance movements revealed related results (Topolinski and Bakhtiari, 2016). The authors investigated the reverse link between affect and movement, that is whether approach movements induce positive affect and avoidance movements induce negative affect. Thereby, they focused on oral approach movements (i.e., oral muscle tensions that wander inward) and oral avoidance movements (i.e., oral muscle tensions that wander outward). Participants were presented with words that featured a sequence of either inward–outward or outward–inward wandering muscle tensions, and were asked to indicate their liking of the words. Participants preferred outward–inward over inward–outward words, suggesting that the final movement more strongly influenced liking than the initial movement.

### Recognizing the Need for a Detour

Recognizing the need for a detour is sometimes easy, other times it requires more complex processing. For instance, if the direct way is blocked by a physical barrier such as a wall, it doesn’t take much to understand that one needs to find a different path. If, however, the direct way is blocked by a closed door, knowing that doors can be opened by turning the doorknob helps to understand that the direct way is still possible. Yet, if the door is locked knowledge about the functioning of locks and keys helps to recognize that one needs to find another way. Cognitive processing is more complex in the latter examples than in the first example, because more input, including knowledge stored in memory, needs to be processed at the same time.

Traditionally, complex processing has often been equated with non-automaticity. However, recent accounts of emotional processing suggest that even complex emotional processes (e.g., constructive appraisals, goal-directed processes) may operate automatically (Moors and De Houwer, 2001; Moors, 2010; Moors et al., 2017). Building on this latter reasoning, the present research investigated whether affective stimuli facilitate ultimately compatible approach–avoidance tendencies, even if relatively complex processing is required to determine the need for a detour. To our knowledge, this is the first study in the domain of approach–avoidance research looking at the complexity of cognitive processing and the immediate-ultimate distinction within one study.

### The Present Research

**FIGURE 1:**
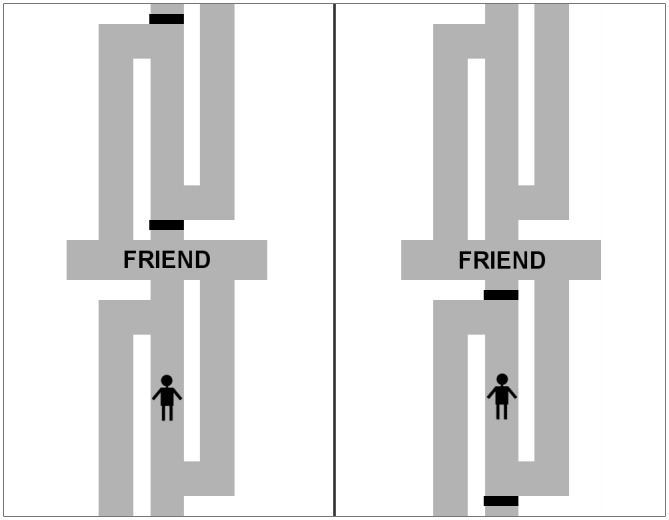
Illustration of the maze presented when recognition of detour was simple. (Left) The direct way was possible. (Right) A detour had to be taken.

**FIGURE 2:**
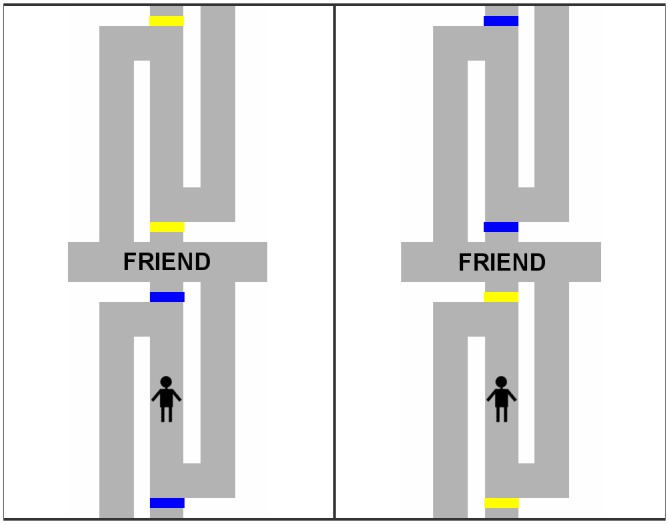
Illustration of the maze presented when recognition of detour was complex. Depending on color condition, the direct way was possible when the bars were blue (left), whereas a detour had to be taken when the bars were yellow (right; or vice versa).

To investigate this question, we followed Krieglmeyer et al. (2011) and adapted the manikin task to manipulate whether the direct way was possible vs. whether a detour was necessary. In particular, the manikin appeared on the middle pathway of a maze (see **Figures 1**, **2**). The middle pathway led from the exit of the maze to the center, where an affective stimulus word was shown. Thus, the middle pathway was the direct way toward or away from the affective stimulus in the center. On half of the trials, the direct way was blocked by a barrier and the manikin had to take a detour by walking along the left or right pathway to reach the center or the exit of the maze. Taking a detour required participants to initially move in the direction opposite to the position ultimately intended.

We manipulated between participants, whether recognizing the need for a detour required simple or more complex cognitive processing. To this end, we manipulated the type of barrier. In the simple condition (**Figure 1**), black solid bars shown across the middle pathway served as barriers. Thus, a single visual cue (i.e., the presence of a black bar) had to be processed to recognize the need for a detour. In the complex condition (**Figure 2**), solid bars were always present but their color (blue vs. yellow) indicated whether they represented locked gates or gates that could be opened. Thus, a property of the visual cue (i.e., the color of the solid bar) had to be processed, and the rule about the meaning of the colors (e.g., blue indicates open; yellow indicates locked) had to be retrieved from short-term memory and had to be applied to the current situation.

In both conditions, participants were asked to respond according to the valence of the stimulus word presented in the center of the maze. In one block of trials, participants were asked to ultimately move the manikin toward positive words and away from negative words (compatible condition). In another block of trials, participants were asked to ultimately move the manikin toward negative words and away from positive words (incompatible condition). Replicating previous research (Krieglmeyer et al., 2011), we expected affective stimuli to facilitate ultimately compatible approach–avoidance behavior in the simple condition. Extending previous research, we tested whether this would also be the case when recognizing the need for a detour involved more complex processing.

## Materials and Methods

### Participants and Design

A total of 87 adults (68 women, 19 men, *M*_age_ = 28.40 years, *SD*_age_ = 10.42 years)^1^ from the participant pool at the University of Würzburg, Germany, participated in exchange for payment. Five participants were excluded because their error rates deviated more than 2.5 SD from the mean error rate, indicating that they had problems following the task instructions (Krieglmeyer et al., 2011).^2^ In previous studies (Krieglmeyer et al., 2011), ultimate compatibility effects were of medium size (mean Cohen’s *d_z_* = 0.68). With about *n* = 40 participants per condition, power was high (*p* = 0.99) in the present experiment (Faul et al., 2007).

The design was a 2 (ultimate compatibility: compatible vs. incompatible) × 2 (way: direct vs. detour) × 2 (recognition of detour: simple vs. complex) mixed within-subjects design with the last variable manipulated between participants.

### Materials

Affective stimuli were 16 positive nouns (e.g., baby, summer, friend) and 16 negative nouns (e.g., death, poison, garbage) selected from Hager and Hasselhorn (1994) and Klauer and Musch (1999). The words appeared in a gray rectangle in the center of a maze (**Figures 1**, **2**). The maze consisted of pathways located above and below the rectangle. We generated two versions of the maze that were flipped along the vertical axis. A manikin was shown on the middle pathway either above or below the word in the center. Furthermore, solid bars were displayed across the middle pathways. Participants were told that the bars were gates.

In the simple condition (**Figure 1**), the gates were painted in black and were displayed only on one pathway, either on the middle pathway above or on the middle pathway below the center. The manikin appeared either between the gates or on the other pathway that contained no gates. Participants were told that the black gates were locked and thus blocked the direct way.

In the complex condition (**Figure 2**), the gates were shown on both middle pathways. The manikin always appeared between gates. The gates on one pathway were painted in blue and the gates on the other pathway were painted in yellow. Participants were told that the blue gates were open and that the yellow gates were locked and, thus, blocked the direct way (or vice versa, counterbalanced across participants).

### Procedure

The procedure was similar to Krieglmeyer et al. (2011). A trial started with the presentation of the maze together with the gates and the manikin. The version of the maze and the location of the gates and the manikin were determined randomly from trial to trial, with the constraint that all possible combinations were displayed equally often. After 750 ms, a stimulus word was presented in the rectangle in the center. Participants were asked to move the manikin toward or away from the word as quickly as possible while making as few errors as possible. To start the movement, they pressed the up or down arrow key once. Then, an animation of the manikin walking to the end position (i.e., the rectangle in the center or the exit of the maze) was shown. The animation lasted 150 ms on the direct way and 250 ms on the detour. Then, the manikin was shown for 100 ms at the end position. The next trial started after 1000 ms. An incorrect response did not start the animation but prompted an error message.

Participants first completed 16 practice trials with a particular response assignment (positive-approach/negative-avoidance vs. negative-approach/positive-avoidance), followed by 64 test trials with the same response assignment. Afterward, they completed 16 practice trials followed by 64 test trials with the reversed response assignment. The order of response assignment was counterbalanced across participants. Demographic information and comments on the task were collected after completion of the task.

## Results

**FIGURE 3:**
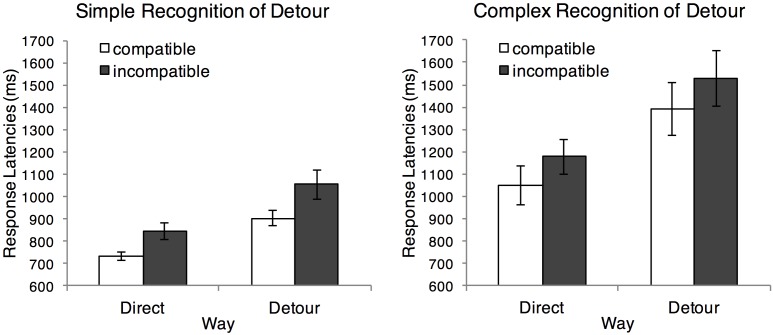
Response latencies as a function of ultimate compatibility (compatible vs. incompatible), way (direct vs. detour), and recognition of detour (simple vs. complex). Error bars represent standard errors of the mean.

As in previous research (Krieglmeyer et al., 2011), we discarded incorrect responses (4.73%) and responses with latencies that deviated more than 2.5 SD from a participant’s individual mean latency in a particular condition (2.54% of the correct responses). ^3^ A 2 (ultimate compatibility: compatible vs. incompatible) × 2 (way: direct vs. detour) × 2 (recognition of detour: simple vs. complex) mixed ANOVA for repeated measures on response latencies revealed a significant main effect of the factor recognition of detour, *F*(1,80) = 17.05, *p* < 0.001, $\eta_{p}^{2}$ = 0.18, indicating that participants responded faster in the simple than in the complex condition (**Figure 3**). A significant main effect of way, *F*(1,80) = 88.36, *p* < 0.001, $\eta_{p}^{2}$ = 0.53, indicated that participants responded faster on direct ways than on detours. Furthermore, the interaction between the factors recognition of detour and way was significant, *F*(1,80) = 7.49, *p* = 0.008, $\eta_{p}^{2}$ = 0.09, suggesting that the response time difference between direct ways and detours was larger in the complex than in the simple condition (all simple comparisons were significant, all *F*s > 15.0, *p*s < 0.001).

Of most interest, the main effect of ultimate compatibility was significant, *F*(1,80) = 11.99, *p* = 0.001, $\eta_{p}^{2}$ = 0.13, indicating that participants responded faster on ultimately compatible trials (*M* = 1014 ms, *SD* = 494 ms) than on ultimately incompatible trials (*M* = 1147 ms, *SD* = 532 ms), Cohen’s *d_z_* = 0.38, 95% CI [57.23 ms, 209.53 ms]. Ultimate compatibility did not interact with the factors recognition of detour and way, all *F*s < 1, *p*s > 0.35. Although ultimate compatibility did not interact with the other factors, we conducted simple comparisons to confirm that the ultimate compatibility effect was significant in all conditions. This was the case. The ultimate compatibility effect was significant in the simple condition on direct ways, *F*(1,80) = 4.75, *p* = 0.032, and on detours, *F*(1,80) = 6.36, *p* = 0.014. Likewise, the ultimate compatibility effect was significant in the complex condition on direct ways, *F*(1,80) = 5.52, *p* = 0.021, and on detours, *F*(1,80) = 4.94, *p* = 0.029.

## Discussion

Participants were faster to ultimately move toward positive and away from negative words than vice versa, even when this required an initial movement in the opposite and thus incompatible direction. These findings replicate the results from previous studies (Krieglmeyer et al., 2011). Most importantly and extending previous research, it did not matter whether recognizing the need for a detour required relatively simple or more complex cognitive processing. More specifically, ultimately compatible approach–avoidance tendencies were triggered to the same extent, when the presence of a simple physical barrier indicated the need for a detour, as well as when the need for a detour had to be inferred from the meaning of the color of gates (i.e., open vs. locked gates). In the latter case, cognitive processing is more complex because in addition to recognizing the color of the gates, the rule about the meaning of the colors has to be retrieved from short-term memory and has to be applied to the current situation. As such, the present findings are in line with current accounts of emotional processing suggesting that even more complex emotional processes can take place automatically (Moors, 2010; Moors et al., 2017).

It is important to note that we successfully manipulated the complexity of the cognitive processing required to recognize the need for a detour, as indicated by the significant effect of the factor recognition of detour. In particular, participants responded more slowly in the complex as compared to the simple condition. In other words, recognizing the need for a detour took more time when the meaning of the barriers had to be inferred from their colors.

### On the Automaticity of the Present Findings

To what extent do the present findings qualify as automatic, i.e., fast, efficient, unintentional/goal-independent, and/or unconscious (Bargh, 1992; Moors and De Houwer, 2006)? In general, results from stimulus-response-compatibility paradigms of approach–avoidance behaviors indicate that the impact of stimulus valence on approach–avoidance tendencies is automatic in the sense of unintentional, i.e., in the absence of the goal to let stimulus valence influence the responses (Krieglmeyer et al., 2013). Thus, we can conclude that affective stimuli trigger ultimately compatible approach–avoidance tendencies in the absence of the goal to let stimulus valence influence the responses. We cannot draw inferences with respect to other features of automaticity, because this would have required additional experimental manipulations. A particularly controversial debate has been centered around the question whether approach–avoidance effects depend on stimulus evaluation goals, with meta-analyses coming to different conclusions (Phaf et al., 2014; Laham et al., 2015; Beatty et al., 2016). However, recent evidence suggests that affective stimuli facilitate even ultimately compatible approach–avoidance tendencies in the absence of evaluation goals (Reichardt, 2018).

### Comparing the Results With the Studies of Krieglmeyer et al. (2011)

Krieglmeyer et al. (2011) conducted two studies with different operationalizations of detours. In Study 1, the manikin appeared between “flying carpets.” Participants moved the manikin on a flying carpet in order to move it toward or away from an affective stimulus. The flying carpets either continued moving in the same direction (= direct way) or moved in the opposite direction (= detour). The color of the carpets indicated how the carpets would move. Participants were faster to move the manikin on that carpet that ultimately moved toward positive and away from negative words than vice versa. Thus, affective stimuli facilitated ultimately compatible approach–avoidance tendencies. However, this effect was significantly smaller on trials with carpets that moved in the opposite direction (i.e., on detours) as compared to trials with carpets that continued moving in the same direction (i.e., on direct ways). This suggests that immediately compatible approach–avoidance tendencies were activated in addition, albeit to a smaller extent than ultimately compatible approach–avoidance tendencies.

In Study 2 of Krieglmeyer et al. (2011), the manikin appeared on a straight vs. winding pathway and participants moved the manikin along the pathway. Straight pathways directly led toward or away from the affective stimulus. Moving on winding pathways implied taking a detour to ultimately move toward or away from the affective stimulus. Again, participants were faster to ultimately move toward positive and away from negative words on straight as well as on winding pathways. Unlike in Study 1, the ultimate compatibility effect did not differ between direct ways and detours. Thus, immediately compatible approach–avoidance tendencies were not activated in this study.

To explain the diverging findings, the authors drew on ideas suggested by Lewin (1935). Lewin assumed that affective stimuli act like forces that directly pull individuals toward them, or push individuals away from them, respectively. In other words, affective stimuli trigger immediately compatible approach–avoidance tendencies. However, if the perception of the situation changes such that the single steps of the detour are represented as a unified action, the first step is no longer perceived as being incompatible. As a consequence, taking a detour becomes easier because affective stimuli no longer trigger immediately compatible approach–avoidance behavior. The layout of the winding pathways in Study 2 of Krieglmeyer et al. (2011) may have facilitated reconstructing the representation of the action sequence as a unified action. As a consequence, immediately compatible approach–avoidance tendencies were no longer activated.

However, the two studies of Krieglmeyer et al. (2011) also differed in the complexity of cognitive processing that was required to recognize the need for a detour. Whereas in Study 1, participants had to infer from the color of the carpets whether the direct way was possible or not, the direct way and the detour were easily recognizable in Study 2 (straight vs. winding pathway). In other words, recognizing the need for a detour required more complex processing in Study 1 than in Study 2. The overall slower responses in Study 1 than in Study 2 support this interpretation. One might argue that immediately compatible approach–avoidance tendencies were not activated in Study 2 because recognizing the need for a detour was easier than in Study 1. In other words, when more complex cognitive processing is required to recognize the need for a detour, as it was the case in Study 1, immediately compatible approach–avoidance tendencies may be activated in the first place.

The present study, however, deems this explanation unlikely. In particular, we did not find any evidence for the activation of immediately compatible approach–avoidance tendencies, neither in the simple nor in the complex condition. That is, because the ultimate compatibility effect did not differ between direct ways and detours, neither in the simple nor in the complex condition. In other words, affective stimuli facilitated ultimately compatible approach–avoidance tendencies to an equal extent, both when recognizing the need for a detour was easy and when this required more complex cognitive processing. Following Lewin (1935) and in line with the reasoning of Krieglmeyer et al. (2011), we suggest that in the present experiment the layout of the pathways facilitated representing the individual steps of the action sequence as a unified action. As a consequence, the first step was no longer perceived as being incompatible with the final direction and, therefore, immediately compatible approach–avoidance tendencies were not activated.

### Implications

A prevalent discussion in the literature on the automatic activation of approach–avoidance tendencies centers around the question whether affective stimuli trigger specific motor patterns of approach–avoidance (e.g., arm flexion and extension) or a rather flexible mechanism of distance regulation (Krieglmeyer et al., 2013; Kozlik et al., 2015). In particular, early studies demonstrated a link between positive valence and arm flexion, and negative valence and arm extension (Cacioppo et al., 1993; Chen and Bargh, 1999; Rotteveel and Phaf, 2004). The authors argued that arm flexion and extension are associated with approach and avoidance because during the lifetime of an organism the activity of these muscles typically co-occurs with approach (pulling positive objects toward) and avoidance (pushing negative objects away), respectively. Later research, however, has shown that the regulation of approach–avoidance behavior is much more flexible. By independently varying the concrete movement (arm flexion vs. extension) and the behavioral effects of the movements (decrease vs. increase of the distance between the self and a stimulus), several researchers showed that affective stimuli facilitate movements that cause a compatible change in distance, regardless of whether the movement involved flexion or extension (Markman and Brendl, 2005; Rinck and Becker, 2007; Seibt et al., 2008). Furthermore, hand postures (hand open vs. closed while holding something) inducing mental simulations of arm movements with different approach–avoidance goals determine whether flexion or extension is associated with approach or avoidance (Freina et al., 2009). Finally, even simple button pressing responses that do not involve arm flexion or extension but lead to the experience of distance change are triggered by affective stimuli (De Houwer et al., 2001; van Dantzig et al., 2008).

Our observation that affective stimuli trigger ultimately compatible approach–avoidance tendencies support the notion of a flexible regulation of distance change. That is, because in the present paradigm neither approach nor avoidance was associated with a specific response (i.e., a specific motor pattern). Instead, on each trial the current context (i.e., the location of the manikin and the type and the location of the barriers) determined which particular response (up or down key press) caused approach or avoidance. Most importantly, by pitting immediate and ultimate distance change effects against each other our findings go beyond previous research by showing that it is the ultimate (not immediate) effect of distance change that matters.

What processes mediate the activation of flexible distance change responses? We suggest that an automatic anticipation of behavioral outcomes in terms of distance change (i.e., being close to or far away from the stimulus) underlies the activation of approach–avoidance tendencies. This reasoning is based on research on non-affective action control (for an overview, see Kunde et al., 2007). In particular, evidence from this field suggests that automatic outcome anticipation mediates response selection and initiation (e.g., Kunde, 2001). According to this research, associations between actions and their perceivable effects are learned through experience. When later a certain effect is desired, the anticipation of the effect automatically activates the action representation that previously produced this effect (ideomotor hypothesis; James, 1890). This mechanism also applies to action-effect association that are not stable but vary depending on the specific context (Kiesel and Hoffmann, 2004). A recent study (Janczyk et al., 2017) even pitted immediate and distal outcomes against each other, as it was done in the present study. The findings from that study indicate that outcome anticipations comprised only representations of distal (but not immediate) outcomes. Taken together, the mechanisms underlying the regulation of approach–avoidance behavior may be the same as the mechanisms underlying the regulation of non-affective behavior, namely anticipation of desired outcomes.

An alternative account on the role of anticipation in the activation of approach–avoidance tendencies focuses on the valence of the behavioral outcomes (Evaluative Coding Account; Lavender and Hommel, 2007; Eder and Rothermund, 2008; Eder and Hommel, 2013). The authors propose that approach responses are represented by positive codes because they produce positive outcomes and avoidance responses are represented by negative codes because they presumably produce negative outcomes. Labeling a response as an approach (or avoidance) response is sufficient to attach a positive (or negative) code to this response, regardless of whether the response actually leads to distance change. From this perspective, compatibility effects in approach–avoidance tasks occur due to an overlap of stimulus valence and response valence. Thus, such effects do not stem from the approach–avoidance nature of the responses. Although we cannot exclude that evaluative coding mechanisms played a role in the present experiment, we deem it unlikely that they fully explain the results because motivational mechanisms of distance change have been shown to underlie effects in the manikin task independent of evaluative coding (Krieglmeyer et al., 2010).

## Conclusion

In sum, the present findings suggest that behavior regulation toward and away from affective stimuli is flexible, relatively farsighted, and automatic to some extent. As such, this mechanism is adaptive because it mobilizes appropriate behavior in situations relevant for survival, growth, and reproduction.

## Ethics Statement

This study was carried out in a manner consistent with the APA’s ethical principles in the conduct of research with human participants.

## Author Contributions

RR planned and conducted the study, analyzed the data, and wrote the manuscript.

## Conflict of Interest Statement

The author declares that the research was conducted in the absence of any commercial or financial relationships that could be construed as a potential conflict of interest.

## Footnotes

1 Note that the majority of the sample is female, as it is often the case in psychological experiments. Even though we are not aware of any research showing gender differences in cognitive processes related to the ones studied in the present experiment, we cannot be sure that our findings generalize to males.

2 Analyses including these participants revealed corresponding results.

3 Analyses, for which we discarded responses with latencies below 150 ms and above 2500 ms, revealed corresponding results.
